# LncRNA EPIC1 downregulation mediates hydrogen peroxide-induced neuronal cell injury

**DOI:** 10.18632/aging.102545

**Published:** 2019-12-08

**Authors:** Jian Sun, Jinyu Zheng, Ya Li, Ming Yan, Ping Li, Lili Ma

**Affiliations:** 1 Department of Anesthesiology, Huai’an Maternity and Child Healthcare Hospital, Yangzhou University Medical School, Huai’an, China; 2 Department of Neurosurgery, The Affiliated Huai’an Hospital of Xuzhou Medical University, Huai’an, China; 3 The Central Lab, North District, Suzhou Municipal Hospital Affiliated to Nanjing Medical University, Suzhou, China; 4 Department of Radiotherapy and Oncology, Affiliated Kunshan Hospital to Jiangsu University, Kunshan, China; 5 Department of Anesthesiology, Jiangsu Cancer Hospital and Jiangsu Institute of Cancer Research and The Affiliated Cancer of Hospital Nanjing Medical University, Nanjing, China

**Keywords:** neuronal cells, hydrogen peroxide, lncRNA EPIC1, MYC

## Abstract

Excessive oxidative stress causes neuronal cell injury. Long non-coding RNA (LncRNA) EPIC1 (Lnc-EPIC1) is a MYC-interacting LncRNA. Its expression and potential functions in hydrogen peroxide (H_2_O_2_)-stimulated neuronal cells are studied. In SH-SY5Y neuronal cells and primary human neuron cultures, H_2_O_2_ downregulated Lnc-EPIC1 and key MYC targets *(Cyclin A1*, *CDC20* and *CDC45*). Ectopic overexpression of Lnc-EPIC1 increased expression of MYC targets and significantly attenuated H_2_O_2_-induced neuronal cell death and apoptosis. Contrarily, Lnc-EPIC1 siRNA potentiated neuronal cell death by H_2_O_2_. MYC knockout by CRISPR/Cas9 method also facilitated H_2_O_2_-induced SH-SY5Y cell death. Significantly, MYC knockout abolished Lnc-EPIC1-induced actions in H_2_O_2_-stimulated neuronal cells. Together, these results suggest that Lnc-EPIC1 downregulation mediates H_2_O_2_-induced neuronal cell death.

## INTRODUCTION

Neurons in the central nerve system (CNS) are vulnerable to reactive oxygen species (ROS) overproduction and excessive oxidative injury. It is possibly due to the high rate of oxygen consumption, enrichment of polyunsaturated fatty acids and defective Nrf2 cascade in neurons [1–5]. Excessive oxidative stress will induce profound neuronal cell injury, serving as a key pathogenesis mechanism of neurodegenerative diseases [1–4]. Hydrogen peroxide (H_2_O_2_), produced during the redox process, has been widely utilized to treat cultured neurons and neuronal cells *in vitro*, mimicking oxidative injury [6–8]. H_2_O_2_ overproduction can induce profound lipid peroxidation, DNA breaks, protein damage and eventually neuronal cell death and apoptosis [7, 9, 10]. Understanding the molecular mechanisms of H_2_O_2_-induced neuronal cell death is vital for developing possible intervention strategies.

Long non-coding RNAs (LncRNAs) are traditionally considered as transcriptional noise. Recently, there are growing literatures showing that LncRNAs are key regulators of almost all cellular and physiological processes [11, 12]. LncRNAs’ functions in CNS and neuronal cells are still largely elusive [13, 14]. The transcription factor MYC is vital for the transcription and expression of key genes of cell survival in neuronal cells [15, 16]. The activity of MYC is mainly controlled by transcriptional and posttranscriptional mechanisms [17, 18]. Wang et al., recently demonstrated that LncRNA EPIC1 (epigenetically-induced LncRNA1, “Lnc-EPIC1”) directly associates to MYC [19].

Lnc-EPIC1 (ENSG00000224271)’s binding to MYC occurs at Lnc-EPIC1’s 129-283 nucleotide region [19]. Lnc-EPIC1 silencing will lead to the inhibited occupancy of MYC to its target genes (*CDC20*, *CDC45* and *CCNA2*) [19], suppressing their expression [19]. Furthermore, MYC knockout abolished Lnc-EPIC1-induced actions [19]. Recently, Zhang et al., have shown that Lnc-EPIC1 protected human osteoblasts from dexamethasone-induced cell death via interacting with MYC [20]. Studies have also shown that Lnc-EPIC1-MYC association promoted survival and growth of human lung cancer cells and cholangiocarcinoma cells [21, 22]. Considering that MYC is important for neuronal survival [15, 16] and Lnc-EPIC1 is vital for its functions [19–22], we here tested expression and potential functions of Lnc-EPIC1 in hydrogen peroxide (H_2_O_2_)-treated neuronal cells.

## RESULTS

### H_2_O_2_ downregulates Lnc-EPIC1 in neuronal cells

**Figure 1:**
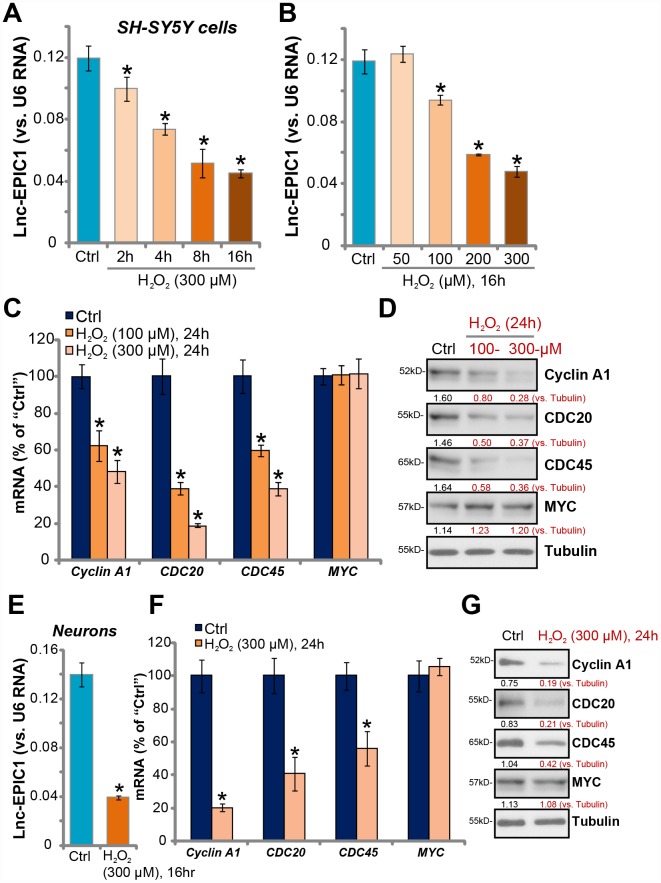
**H_2_O_2_ downregulates Lnc-EPIC1 in neuronal cells.** SH-SY5Y cells (**A**–**D**) or the primary human neuron cultures (**E**–**G**) were treated with hydrogen peroxide (H_2_O_2_, 50-300 μM), cells were further cultured for indicated time periods, expression of Lnc-EPIC1 (**A**, **B** and **E**, vs. *U6 RNA*) and listed mRNAs (**C** and **F**, vs. *GAPDH*) were tested by qPCR assay. Listed proteins in total cell lysates were tested by Western blotting assay (**D** and **G**). Listed proteins were quantified, with the values normalized to Tubulin (**D** and **G**). “Ctrl” stands for untreated control cells (same for all Figures). “LDH%” stands for medium LDH ratio, indicating cell death (same for all Figures). Bars stand for mean ± standard deviation (SD, n=5). * *P* < 0.05 *vs.* “Ctrl” cells. Experiments in this figure were repeated three times, and similar results were obtained.

In order to study the potential effect of LncRNA EPIC1 (Lnc-EPIC1) in neuronal cells, differentiated SH-SY5Y neuronal cells were treated with H_2_O_2_. Expression of Lnc-EPIC1 was tested by qPCR assay using the described primers [21, 22]. Its level was normalized to *U6 RNA*. As demonstrated, H_2_O_2_ treatment (300 μM) time-dependently decreased Lnc-EPIC1 level in SH-SY5Y cells (Figure 1A). Its levels reduced to 83.51 ± 6.56%, 61.79 ± 3.31%, 43.34 ± 7.72%, 37.84 ± 2.10% of control after 2h, 4h, 8h and 16h of H_2_O_2_ treatment, respectively (Figure 1A). Further, in a dose-dependent manner H_2_O_2_ downregulated Lnc-EPIC1 in SH-SY5Y cells (Figure 1B). Lnc-EPIC1 downregulation was significant following 100-300 μM of H_2_O_2_ treatment (Figure 1B). At the lowest concentration (50 μM) H_2_O_2_ failed to significantly alter Lnc-EPIC1 expression (*P* > 0.05 *vs.* “Ctrl”, Figure 1B).

Lnc-EPIC1 directly interacts with MYC, essential for MYC function and expression of key MYC targets [19, 21, 22]. qPCR assay results, in Figure 1C, demonstrated that mRNA levels of the MYC targets, *Cyclin A1*, *CDC20* and *CDC45* [19, 21, 22], were significantly downregulated after H_2_O_2_ (100/300 μM) treatment in SH-SY5Y cells. Cyclin A1, CDC20 and CDC45 protein levels were decreased as well (Figure 1D), where MYC mRNA and protein expression were unchanged (Figure 1C and 1D).

In the primary human neuronal cultures, H_2_O_2_ treatment (300 μM, 16h) significantly downregulated Lnc-EPIC1 expression (28.24 ± 1.21% of control, Figure 1E). mRNA and protein expression levels of MYC targets, Cyclin A1, CDC20 and CDC45, were also downregulated (Figure 1F and 1G). MYC expression was again unchanged by H_2_O_2_ (Figure 1F and 1G). These results show that H_2_O_2_ downregulates Lnc-EPIC1 and MYC targets in neuronal cells, indicating a functional activity of Lnc-EPIC1 in H_2_O_2_-induced neuronal cytotoxicity.

### Ectopic overexpression of Lnc-EPIC1 inhibits H_2_O_2_-induced neuronal cytotoxicity

**Figure 2:**
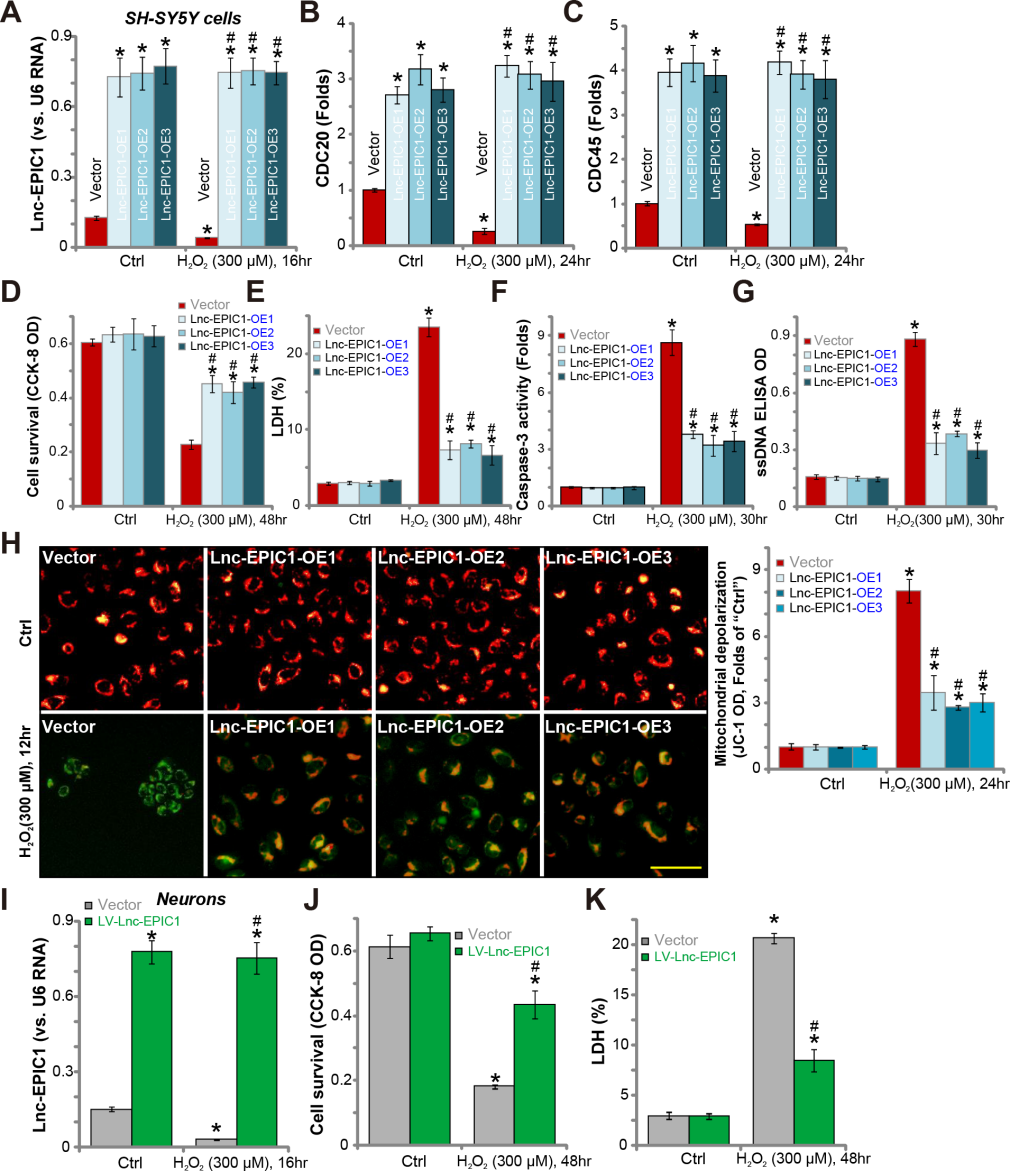
**Ectopic overexpression of Lnc-EPIC1 inhibits H_2_O_2_-induced neuronal cytotoxicity.** Stable SH-SY5Y cells with the lentiviral Lnc-EPIC1-expression construct (three lines, “Lnc-EPIC1-OE-1/-2/-3”) or the vector control cells (“Vector”) were treated with hydrogen peroxide (H_2_O_2_, 300 μM), cells were further cultured for indicated time, expression of Lnc-EPIC1 (**A**) and listed mRNAs (**B** and **C**) were tested by qPCR assay; Cell survival (by the CCK-8 assay, **D**), death (by the LDH assay, **E**) and apoptosis (by the caspase-3 activity, ssDNA ELISA and JC-1 staining assays, **F**–**H**) were tested. The primary human neuron cultures were infected with the lentivirus with Lnc-EPIC1 construct (“LV-Lnc-EPIC1”) or empty vector (“Vector”) for 48h, treated with hydrogen peroxide (H_2_O_2_, 300 μM) for applied time, Lnc-EPIC1 expression (**I**), neuronal survival (by the CCK-8 assay, **J**) and death (by the LDH assay, **K**) were tested. Bars stand for mean ± standard deviation (SD, n=5). * *P* < 0.05 *vs.* “Ctrl” treatment of “Vector” cells. ^#^ *P* < 0.05 *vs.* H_2_O_2_ treatment of “Vector” cells. Experiments in this figure were repeated three times, and similar results were obtained. Bar= 100 μm (**H**).

In order to test the potential function of Lnc-EPIC1 in H_2_O_2_-induced neuronal cytotoxicity, a lentiviral Lnc-EPIC1-expression construct was transfected to SH-SY5Y cells. Three stable SH-SY5Y cell lines (“Lnc-EPIC1-OE-1/-2/-3”) were established following puromycin selection (see Methods). qPCR assay was performed to show that Lnc-EPIC1 levels were significantly elevated in the Lnc-EPIC1-OE cells, with/without H_2_O_2_ treatment (Figure 2A). Expression of key MYC targets, including CDC20 and CDC45, were dramatically increased in Lnc-EPIC1-OE cells (Figure 2B and 2C). Further, H_2_O_2_-induced downregulation of CDC20 and CDC45 was reversed by Lnc-EPIC1 overexpression (Figure 2B and 2C).

Significantly, H_2_O_2_-induced cell viability (CCK-8 OD) reduction (Figure 2D) and cell death (medium LDH release, Figure 2E) were largely attenuated by Lnc-EPIC1 overexpression (Figure 2D and 2E). H_2_O_2_ induced significant apoptosis activation in control SH-SY5Y cells, evidenced by caspase-3 activation (Figure 2F) and single-strand DNA (“ssDNA”) accumulation (Figure 2G), which were significantly attenuated in Lnc-EPIC1-OE cells (Figure 2F and 2G). Furthermore, H_2_O_2_-induced mitochondrial depolarization, evidenced by JC-1 green fluorescence accumulation, was largely inhibited with Lnc-EPIC1 overexpression (Figure 2H).

In primary human neuron cultures, transfection of the lentiviral Lnc-EPIC1-expression construct (“LV-Lnc-EPIC1”) significantly increased Lnc-EPIC1 expression, even after H_2_O_2_ stimulation (Figure 2I). H_2_O_2_-induced neuronal death, reflected by CCK-8 OD reduction (Figure 2J) and medium LDH release (Figure 2K), was significantly attenuated by LV-Lnc-EPIC1 (Figure 2I–2K). Collectively, these results show that ectopic overexpression of Lnc-EPIC1 attenuates H_2_O_2_-induced neuronal cytotoxicity.

### Lnc-EPIC1 siRNA potentiates H_2_O_2_-induced neuronal cell death

**Figure 3:**
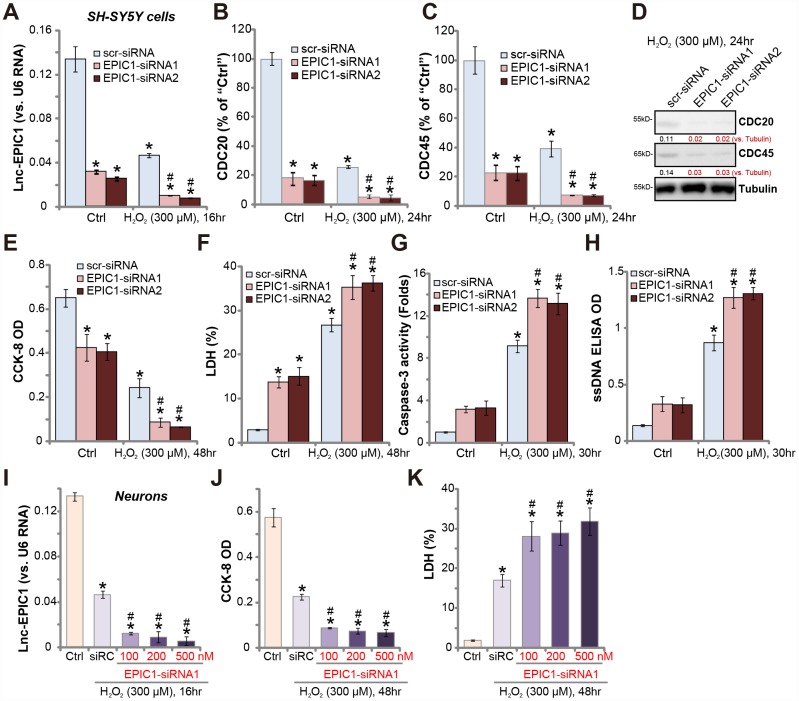
**Lnc-EPIC1 siRNA potentiates H_2_O_2_-induced neuronal cell death.** SH-SY5Y cells (**A**–**H**) or the primary human neuron cultures (**I**–**K**) were transfected with applied Lnc-EPIC1 siRNA (“EPIC1-siRNA1/2”, 100-500 nM) or scramble control siRNA (“scr-siRNA”, 500 nM) for 48h, then treated with/without hydrogen peroxide (H_2_O_2_, 300 μM), cells were further cultured for indicated time, expression of Lnc-EPIC1 (**A** and **I**) and listed mRNAs (**B** and **C**) were tested by qPCR assay; Expression of listed proteins was tested by Western blotting assay (**D**); Cell viability (by the CCK-8 assay, **E** and **J**), cell death (by the LDH assay, **F** and **K**) and apoptosis (by the caspase-3 activity and ssDNA ELISA, **G** and **H**) were tested. Listed proteins were quantified, with the values normalized to Tubulin (**D**). Bars stand for mean ± standard deviation (SD, n=5). * *P* < 0.05 *vs.* “Ctrl” treatment in “scr-siRNA” cells. ^#^ *P* < 0.05 *vs.* H_2_O_2_ treatment of “scr-siRNA” cells. Experiments in this figure were repeated three times, and similar results were obtained.

Since Lnc-EPIC1 overexpression protected neuronal cells from H_2_O_2_ (see Figure 2), Lnc-EPIC1 silence should then potentiate H_2_O_2_-induced neuron injury. To test this hypothesis, two different Lnc-EPIC1 siRNAs (“EPIC1-siRNA1/2”), with non-overlapping sequences [19, 21, 22], were transfected to SH-SY5Y cells. As demonstrated, each of the applied EPIC1-siRNA (at 500 nM) further decreased Lnc-EPIC1 level in H_2_O_2_-treated SH-SY5Y cells (Figure 3A). MYC targets, including *CDC20* and *CDC45*, were downregulated by EPIC1-siRNA (Figure 3B and 3C). There levels went further decreased following H_2_O_2_ treatment (Figure 3B and 3C). CDC20 and CDC45 protein levels were also decreased in Lnc-EPIC1-silneced SH-SY5Y cells after H_2_O_2_ treatment (Figure 3D).

Importantly, in SH-SY5Y cells, H_2_O_2_-induced viability (CCK-8 OD) reduction (Figure 3E), cell death (medium LDH release, Figure 3F) and apoptosis (increases in caspase-3 activity and ssDNA accumulation, Figure 3G and 3H) were significantly potentiated by Lnc-EPIC1 siRNAs (Figure 3E–3H). Lnc-EPIC1 siRNA alone also induced significant SH-SY5Y cell death and apoptosis (Figure 3E–3H).

In the primary human neuronal cultures, siRNA-mediated knockdown of Lnc-EPIC1 (by “EPIC1-siRNA1”, see Figure 3I) augmented H_2_O_2_-induced viability reduction (Figure 3J) and cell death (Figure 3K). Collectively, H_2_O_2_-induced neuronal cytotoxicity is exacerbated by Lnc-EPIC1 silencing, further supporting that Lnc-EPIC1 downregulation mediates H_2_O_2_-induced cytotoxicity in the neuronal cells.

### MYC knockout abolishes Lnc-EPIC1-induced actions in H_2_O_2_-stimulated neuronal cells

**Figure 4:**
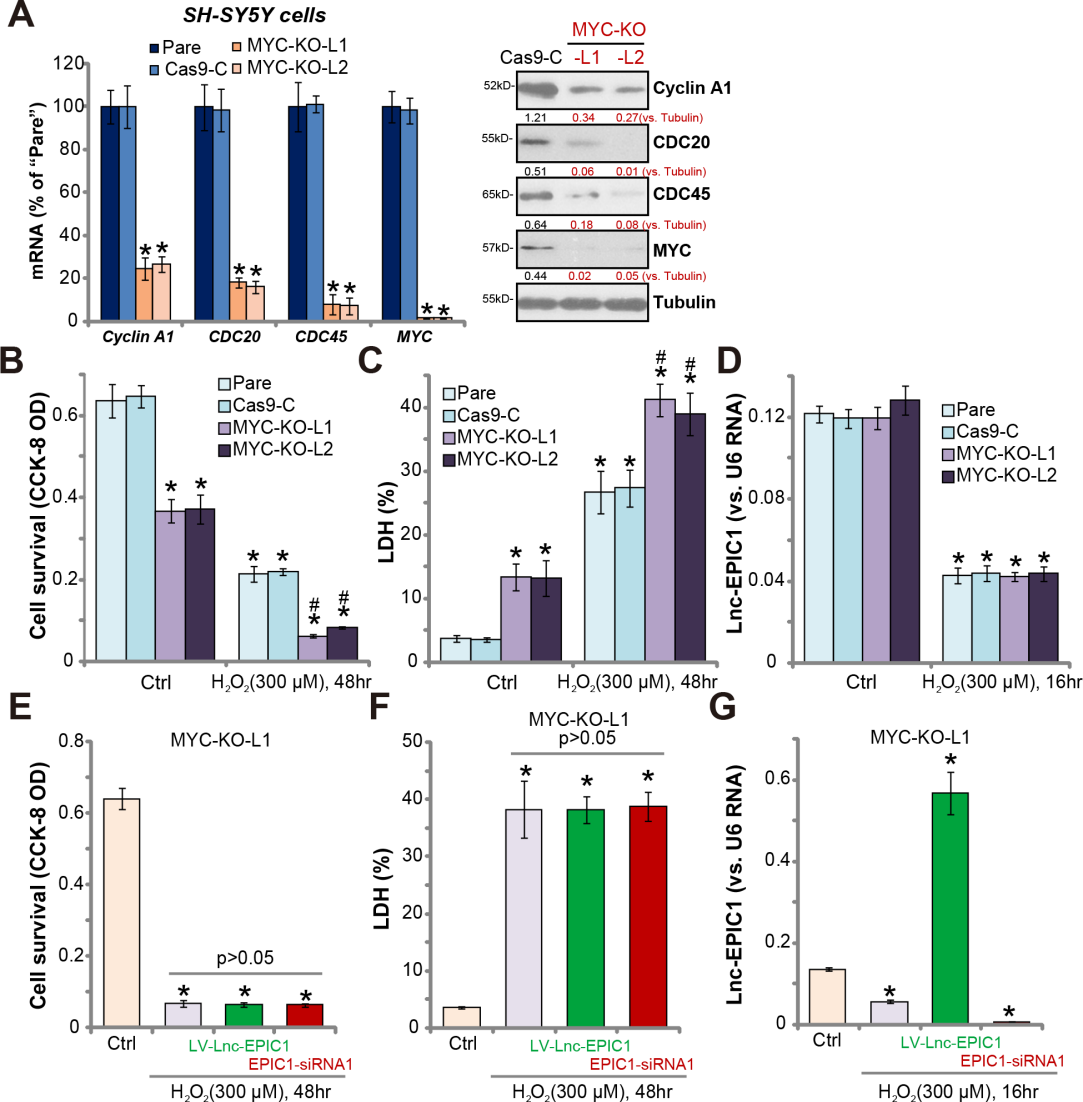
**MYC knockout abolishes Lnc-EPIC1-induced actions in H_2_O_2_-stimulated neuronal cells.** Expression of listed mRNAs and proteins in the stable SH-SY5Y cells with the lenti-CRISPR/Cas9-MYC-KO-GFP constructs (“MYC-KO-L1/L2”) or the control construct (“Cas9-C”) as well as in the parental control cells (“Pare”) were shown (**A**). Above cells were treated with/without hydrogen peroxide (H_2_O_2_, 300 μM) for the applied time, cell viability (by CCK-8 assay, **B**) and death (by LDH assay, **C**) were examined; Lnc-EPIC1 levels were tested by qPCR assay (**D**). “MYC-KO-L1” cells were transfected with lentiviral Lnc-EPIC1 construct (“LV-Lnc-EPIC1”) or Lnc-EPIC1 siRNA-1 (“EPIC1-siRNA1”, 500 nM) for 48h, followed by hydrogen peroxide (H_2_O_2_, 300 μM) stimulation for the applied time, cell viability (**E**), cell death (**F**) and Lnc-EPIC1 expression (**G**) were tested. Listed proteins were quantified, with the values normalized to Tubulin (**A**). Bars stand for mean ± standard deviation (SD, n=5). * *P* < 0.05 *vs.* “Ctrl” treatment of “Pare” cells (**A**–**D**). * *P* < 0.05 *vs.* “Ctrl” treatment (**E**–**G**). ^#^ *P* < 0.05 *vs.* H_2_O_2_ treatment of “Pare” cells (**A**–**C**). Experiments in this figure were repeated three times, and similar results were obtained.

If MYC is the primary target of Lnc-EPIC1 in neuronal cells, Lnc-EPIC1 should be ineffective in the MYC-knockout cells. To test this hypothesis, CRISPR/Cas9 strategy was applied. Two different lenti-CRISPR/Cas9-MYC-KO-GFP constructs, targeting non-overlapping DNA sequence of MYC, were transfected to SH-SY5Y neuronal cells. Via FACS-mediated GFP sorting and puromycin selection, stable monoclonal cells were established (“MYC-KO-L1/L2”), showing completely depleted *MYC mRNA* (Figure 4A). Consequently, mRNA expression of MYC targets, including *Cyclin A1*, *CDC20* and *CDC45*, were significantly downregulated (Figure 4A, left panel). Additionally, cyclin A1, CDC20 and CDC45 protein levels were largely reduced in MYC-KO SH-SY5Y cells (Figure 4A, right panel).

MYC-KO cells presented with decreased cell viability and increased cell death, when compared to the control cells with lenti-CRISPR control construct (“Cas9-C”) (Figure 4B and 4C). Importantly, MYC-KO cells were more vulnerable to H_2_O_2_, by showing significantly enhanced viability reduction and death versus control cells (Figure 4B and 4C). Therefore, MYC-KO cells presented with similar phenotypes to the Lnc-EPIC1-silenced cells (Figure 3). Notably, MYC-KO did not significantly change Lnc-EPIC1 expression in SH-SY5Y cells with/without H_2_O_2_ stimulation (Figure 4D).

Further studies demonstrated that in MYC KO cells (“MYC-KO-L1”), Lnc-EPIC1 overexpression (by “LV-Lnc-EPIC1”) or silencing (by “EPIC1-siRNA1”) failed to change H_2_O_2_-induced cytotoxicity (Figure 4E and 4F). Although both LV-Lnc-EPIC1 or the siRNA altered Lnc-EPIC1 expression in H_2_O_2_-stimulated neuronal cells (Figure 4G). Similar results were also obtained in the “MYC-KO-L2” cells (Data not shown). Therefore, MYC knockout abolished Lnc-EPIC1-induced actions in H_2_O_2_-stimulated SH-SY5Y cells, suggesting that MYC is the primary target of Lnc-EPIC1.

## DISCUSSION

LncRNAs are involved in regulating almost all key cellular behaviors, by functioning as molecular signals, guides, scaffolds, enhancers or microRNA spongers, and altering gene transcription and/or functions [11, 12, 23]. Moreover, LncRNAs are important for genomic imprinting, cell cycle progression, cell survival, differentiation and development [11, 12, 23]. A growing number of studies have reported that aberrant LncRNA expression is detected in neuronal cells and human brain, participating in the pathogenesis of brain and even in neurodegenerative diseases [14]. LncRNA can function as a endogenous RNA (ceRNA) for various miRNAs to inhibit or promote neuronal cell apoptosis. For example, Li et al., have shown that LncRNA KCNQ1OT1 promoted SH-SY5Y neuronal cell apoptosis by sponging miR-296 and upregulating Bax [24]. In ischemic stroke LncRNA SNHG6 (small nucleolar RNA host gene 6) promoted neuronal cell apoptosis by inhibiting miR-181c but upregulating its target Bim [25]. Here we tested the involvement of Lnc-EPIC1 in H_2_O_2_-induced neuronal cell death.

LncRNA-mediated regulation on MYC transcriptional activity is largely unknown until recently [19]. MYC by itself is unable to form a homodimer nor binding to DNA. Certain LncRNAs interact directly with proteins and can influence the structural state and activity of these proteins [14]. Wang et al., have demonstrated that Lnc-*EPIC1* regulates MYC’s occupancy on several key MYC target genes, including *Cyclin A1*, *CDC20* and *CDC45* [19]. By directly binding to double-strand DNA, Lnc-*EPIC1* functions as the “guide” RNA to promote expression of MYC targets [19]. Furthermore, MYC-MAX association can also be facilitated by Lnc*-EPIC1 [19]. Here our evidence suggest that* Lnc-EPIC1 is also vital for the survival of neuronal cells.

We here reported a new mechanism of Lnc-EPIC1 in promoting neuronal cell survival via directly interacting with MYC, and Lnc-EPIC1 is important for MYC function in neuronal cells. In both SH-SY5Y cells and primary neuron cultures, forced overexpression of Lnc-EPIC1 significantly increased mRNA and protein expression of key MYC targets, including *Cyclin A1*, *CDC20* and *CDC45*. Contrarily, their levels were significantly reduced by siRNA-mediated silencing of Lnc-EPIC1. Importantly, Lnc-EPIC1 was decreased in H_2_O_2_-treated SH-SY5Y cells and primary neuronal cultures. Therefore, H_2_O_2_ downregulates Lnc-EPIC1 to inhibit MYC functions, which could be an important mechanism responsible for H_2_O_2_-induced neuronal cell death.

A number of recent data have implied that Lnc-EPIC1, by directly interacting with MYC, promotes survival of human cells [20–22]. However, Zhao et al., have reported that it inhibited osteosarcoma cell survival by inducing MEF2D ubiquitination [26]. Results from this study support that Lnc-EPIC1 exerts neuronal protective functions. We show that Lnc-EPIC1 downregulation mediated H_2_O_2_-induced neuronal cytotoxicity. Importantly, ectopic overexpression of Lnc-EPIC1 using a lentiviral construct significantly attenuated H_2_O_2_-induced neuronal cytotoxicity. On the contrary, siRNA-mediated silencing of Lnc-EPIC1 potentiated neuronal cell death and apoptosis by H_2_O_2_. Lnc-EPIC1 siRNA alone was cytotoxic to neuronal cells as well. Thus, targeting Lnc-EPIC1 could be a novel strategy for neuronal protection against oxidative injury.

MYC is a transcription factor regulating expression of a wide variety of genes of cell survival. In the present study, we show that MYC KO by CRISPR/Cas9 method induced neuronal cell death and apoptosis. MYC KO also intensified H_2_O_2_-induced neuronal cytotoxicity. Importantly, neither Lnc-EPIC1 overexpression nor Lnc-EPIC1 siRNA was functional in MYC-KO cells. These evidence indicated that MYC is the primary target of Lnc-EPIC1 in neuronal cells. Further insight studies are necessary to explore the interaction between Lnc-EPIC1 and MYC as well as their functions in neuronal cells. In summary, we conclude that Lnc-EPIC1 downregulation mediates H_2_O_2_-induced neuronal cell death.

## MATERIALS AND METHODS

### Reagents

H_2_O_2_, puromycin, polybrene, fetal bovine serum (FBS) and other cell culture reagents were provided by Sigma-Aldrich Co. (St. Louis, MO). Antibodies were purchased from Cell Signaling Technology Co. (Shanghai, China). mRNA primers and sequences were synthesized by Genechem (Shanghai, China). All the transfection reagents were purchased from Invitrogen-Thermo Fisher Co. (Shanghai, China).

### SH-SY5Y cells

Human neuronal SH-SY5Y cells (from Dr. Gao [27]) were cultured in DMEM plus 10% FBS. Before H_2_O_2_ treatment, SH-SY5Y cells were cultured for 10 days in retinoic acid (RA, 10 μM)-containing complete DMEM medium, followed by another five days culture in serum-free DMEM with BDNF (50 ng/mL) and glutamine (2 mM).

### Primary human neuron cultures

The primary human neuron cultures were provided by Dr. Zhang [28]. Briefly, the normal fetal brains were digested and filtered. The dissociated neurons were centrifuged and resuspended in MEM medium with the described supplements [29]. Neurons were plated in poly-L-lysine-coated six-well plates. Astrocytes were abandoned [29]. Neuron cultures were grown for eight days (day *in vitro* 8, DIV8) before any further experiments. The protocols of using human cells were approved by the Ethics Committee of authors’ institutions.

### Quantitative real-time PCR assay (qPCR)

Following the applied treatment, the TRIzol reagents (Promega) was applied to extract total cellular RNA. The Fast-Start Universal SYBR Green Master mix (Roche) was used for the qPCR. Melting curve analysis was performed to calculate the product melting temperature. qPCR quantification was through 2^—ΔCt^ method using the following formula: 2^—(Ct of target gene—Ct of reference gene)^. The data presented were normalized to *GAPDH* transcripts. mRNA primers for *MYC, Cyclin A1,* *CDC20*, and *CDC45* [19] and Lnc-EPIC1 [22] were synthesized by Genechem (Shanghai, China). Lnc-EPIC1 expression was normalized to *U6 RNA.*

### Western blotting assay

For each treatment, 40 μg protein lysates were separated by 10% SDS-PAGE gels, transferred to PVDF blots. After blocking in 10% non-fat milk, the blots were probed with the appropriate primary antibodies, followed by incubation in the HRP-conjugated secondary antibodies. The blots were visualized by the ECL system (Sigma). Quantification of the band intensity was performed via using the Image J software.

### Ectopic Lnc-EPIC1 overexpression

The pLenti6-GFP-puro expression vector with Lnc-EPIC1 sequence (“LV-EPIC1”) was provided by Dr. Zhang [22], transfected to SH-SY5Y cells (in polybrene-containing medium) through Lipofectamine 2000. Stable cells were selected via puromycin (2.0 μg/mL). Three stable SH-SY5Y cell lines with LV-EPIC1 were established, named as “Lnc-EPIC1-OE1/2/3”. qPCR assay was performed to verify Lnc-EPIC1 overexpression. Control cells were transfected with empty vector (“Vector”). For the primary human neuron cultures, LV-EPIC1 was directly transfected to neurons, and no puromycin-mediated selection was performed.

### Cell viability assay

Neuronal cells were seeded in the 96-well plates (2 × 10^4^ cells/cm^2^). Following treatment, cell viability was examined by the Cell Counting Kit-8 (CCK-8, Dojindo, Japan). CCK-8 optical density (OD) values at 550 nm were recorded.

### LDH assay

Following the applied treatment, lactate dehydrogenase (LDH) release to the conditional medium of dying cells was tested by a two-step LDH detection kit (Takara, Tokyo, Japan). Medium LDH was normalized to total LDH.

### Lnc-EPIC1 siRNA

Two Lnc-EPIC1 siRNAs, 5′-CCUUCAGACUGUCUUUGAA-3′ (“EPIC1-siRNA1”) and 5′-GCUUUCUCUCGGAAACGUG-3′ (““EPIC1-siRNA2”), were utilized [21, 22]. SH-SY5Y cells or the primary neurons were seeded in the six well tissue culture plates (2 × 10^4^ cells/cm^2^), transfected with Lnc-EPIC1 siRNA or the scramble control siRNA (“scr-siRNA”) using the Lipofectamine RNAiMAX Reagent for 48h. Lnc-EPIC1 knockdown was verified by qPCR assay.

### Caspase-3 activity assay

The caspase-3 activity assay was described in detail elsewhere [30].

### ssDNA ELISA assay

Single strand DNA (ssDNA) contents were measured as a characteristic marker of cell apoptosis. In brief, thirty μg cell lysates per treatment were analyzed by the ssDNA ELISA (Roche, Basel, Switzerland) to quantify DNA fragmentations. The ssDNA ELISA absorbance values were recorded at 405 nm.

### JC-1 assay of mitochondrial depolarization

JC-1, a fluorescence dye, can form green monomers and aggregate in mitochondria in stressed cells with mitochondrial depolarization [31]. Following the applied H_2_O_2_ treatment, neuronal cells were first stained with JC-1 (10 μg/mL), washed and tested immediately via a fluorescence spectrofluorometer at 550 nm. The representative JC-1 images, integrating the green (at 550 nm) and red (at 625 nm) fluorescence wavelength were presented.

**MYC knockout**

MYC small guide RNA (sgRNA) (sgRNA-1: 5′-GCCGTATTTCTACTGCGACG-3′ [22] or sgRNA-2: 5′-CTATGACCTCGACTACGACT-3′) was packed into the lenti-CRISPR-GFP vector (From Dr. Zhang [22]). By using Lipofectamine 2000 each of the two constructs was individually transfected to SH-SY5Y cells. GFP-positive cells were FACS sorted. After culturing for another 3 weeks, cells were distributed to six well plates, followed by genotyping of depleted MYC. The monoclonal cells were further selected by puromycin for seven days. Control cells were transfected with lenti-CRISPR/Cas9-GFP construct with scramble sgRNA (“Cas9-c”).

### Statistical analysis

Data were expressed as the mean ± standard deviation (SD). Statistical analyses among different groups were performed by the one-way analysis of variance (ANOVA) with Scheffe’s test using SPSS18.0 software (SPSS Inc., Chicago, IL). The two-tailed paired T tests (Excel 2007) were calculated to compare significance between two groups. Values of *P* < 0.05 were considered statistically significant. Experiments were repeated at least three times.
